# Treatment of Cystitis

**Published:** 1881-01

**Authors:** 


Treatment of Cystitis.—In an article upon cystitis, published in the *Dictionaire Encyclopedique*, M. Chauvel recalls the advice given by M. Diday in the treatment of inflammation of the neck of the bladder. Drink three or four times a day an infusion of linseed mixed with pearl barley; rub into the lumbar region antimonial ointment until an eruption takes place; resist the desire to evacuate the last drops of urine, and take three times a day one of the following powders: Sugar in powder, ℥ ss.; hyoscyamus leaves in powder, ʒ ss.; divide into 20 powders. If the pain persisted, M. Chauvel would give every half hour a tablespoonful of an infusion of 45 grains of hyoscyamus leaves in ℥ iv. of water. In a few hours there is almost always relief. If the toxic effect of the medicine manifested itself, coffee was the antidote. In chronic cystitis, the author obtained good results from the following preparation, of which about the size of a cherry-stone was given morning and evening, in a wafer: Turpentine, ʒ ss.; camphor, gr. xv,; ext. hyoscyamus, gr. iij.—*Phila. Med and Surg. Reporter.*

